# Synthetic Microbial Community Members Interact to Metabolize Caproic Acid to Inhibit Potato Dry Rot Disease

**DOI:** 10.3390/ijms25084437

**Published:** 2024-04-18

**Authors:** Huiqin Shi, Wei Li, Hongyu Chen, Yao Meng, Huifang Wu, Jian Wang, Shuo Shen

**Affiliations:** 1 Academy of Agriculture and Forestry Sciences, Qinghai University, Xining 810016, China; shq18822728027@163.com (H.S.); lwbabylw@163.com (W.L.); hongyuchenred@163.com (H.C.); mygzbj@163.com (Y.M.); wuhuifang678@163.com (H.W.); jianwang2197@163.com (J.W.); 2 Key Laboratory of Potato Breeding of Qinghai Province, Xining 810016, China; 3 State Key Laboratory of Plateau Ecology and Agriculture, Qinghai University, Xining 810016, China; 4 Key Laboratory of Qinghai Tibet Plateau Biotechnology, Ministry of Education, Xining 810016, China; 5 Northwest Potato Engineering Research Center, Ministry of Education, Xining 810016, China

**Keywords:** synthetic microbial community, potato dry rot, *Fusarium solani*, caproic acid

## Abstract

The potato dry rot disease caused by *Fusarium* spp. seriously reduces potato yield and threatens human health. However, potential biocontrol agents cannot guarantee the stability and activity of biocontrol. Here, 18 synthetic microbial communities of different scales were constructed, and the synthetic microbial communities with the best biocontrol effect on potato dry rot disease were screened through in vitro and in vivo experiments. The results show that the synthetic community composed of *Paenibacillus amylolyticus*, *Pseudomonas putida*, *Acinetobacter calcoaceticus*, *Serratia proteamaculans*, *Actinomycetia bacterium* and *Bacillus subtilis* has the best biocontrol activity. Metabolomics results show that *Serratia protoamaculans* interacts with other member strains to produce caproic acid and reduce the disease index to 38.01%. Furthermore, the mycelial growth inhibition after treatment with caproic acid was 77.54%, and flow cytometry analysis showed that the living conidia rate after treatment with caproic acid was 11.2%. This study provides potential value for the application of synthetic microbial communities in potatoes, as well as the interaction mechanisms between member strains of synthetic microbial communities.

## 1. Introduction

Potato (*Solanum tuberosum* L.) is a food crop for human consumption, with enormous potential in terms of food security [1]. However, the outbreak of pathogenic microbial diseases has led to the infection of potatoes with various diseases [2,3]. Among them, dry rot is one of the common diseases of potatoes worldwide [4,5]. The losses caused by potato dry rot disease account for over 88% and 50% of the total post-harvest losses in some regions of China and the United States, respectively [6]. 

*Fusarium* spp. will cause various crops to be affected by diseases, including potatoes, wheat, and rice [7,8]. More than 13 species of *Fusarium* spp. cause potato dry rot, including *Fusarium sambucinum*, *F. solani*, *F. graminearum*, *F. oxysporum* et al. [5,9,10,11]. The common method to prevent the spoilage and disease development of *Fusarium* spp. is to immerse the potato tubers in a fungicide suspension before storage. However, this strategy can lead to environmental pollution, chemical toxicity to humans, animals, and the development of resistance to *Fusarium* spp. [12]. Therefore, in order to reduce the negative impact of fungicides, it is necessary to find a new sustainable environmental control strategy [13,14,15]. Biocontrol is an environmental protection strategy for sustainable agricultural development and deserves widespread development [16,17]. For example, *Pseudomonas putida* aggregates around the host wound to form a biofilm, thereby exhibiting antibacterial activity against various plant pathogens [18,19]. And *Serratia marcescens* has been reported to have biocontrol effects on nematodes and mold [20,21]. Furthermore, lipopeptides itrin A and fengycin A produced by *Bacillus* can inhibit the growth of mycelium infected with late blight in vitro and reduce the incidence rate of potato late blight [16]. And the volatile organic compounds released by *Trichoderma* can also inhibit the mycelial growth of late blight disease [22]. In addition, the caproic acid produced by lactic acid bacteria is a key substance that inhibits pathogenic fungi by increasing the permeability of the pathogen’s cell membrane and releasing the electrolyte and protein inside the pathogen’s cells [23]. Furthermore, the secretion of acetic acid, propionic acid, and caproic acid by *B. megaterium*, *B. subtilis*, and *B. simplex* can enhance the phosphorus increasing ability of crops, promote seed germination and nutrient growth [24]. However, currently, single strain biocontrol measures face a situation of low activity and poor stability [14,25,26]. 

Recently, research has found that treatment with multiple microorganisms significantly reduces disease index and enriches potential beneficial microorganisms [17]. Multiple microbial combinations indirectly secrete organic acids such as lactic acid and caproic acid, thereby promoting the growth and metabolism of rhizosphere microorganisms [27]. Furthermore, a core community composed of five bacteria can protect the host from pathogen invasion by producing antifungal compounds surfactin and siderophores from different members [14]. In addition, the co-cultivation of *Streptomyces coelicolor* and *B. subtilis* increased the yield of undecylprodigiosin, thereby inhibiting the fungal pathogen *Verticillium dahliae* [13]. However, the inhibition of potato dry rot during storage by synthesizing microbial communities still needs to be verified and investigated. And the mechanism by which synthetic microbial communities inhibit *Fusarium* sp. is still unclear.

In this study, we isolated strains from potatoes and constructed 18 synthetic microbial communities of different scales. Screening the best synthetic microbial community for inhibiting potato dry rot disease was performed through in vivo and in vitro experiments. Simultaneously, we explored key strains in the synthetic microbial community. Then, key metabolites in the synthetic microbial community were found through metabolomics, and active metabolites were validated. Finally, the mechanism by which synthetic microbial communities inhibit *Fusarium solani* was analyzed through flow cytometry and fluorescence microscopy. This study provides potential value for the application of synthetic microbial communities in potatoes, as well as the interaction mechanisms between member strains of synthetic microbial communities. Furthermore, it provides a new perspective on the problem of low inhibitory activity faced by single strain biocontrol.

## 2. Results and Discussion

### 2.1. Isolation and Identification of Strains That Inhibit Fusarium *spp.*

**Figure 1:**
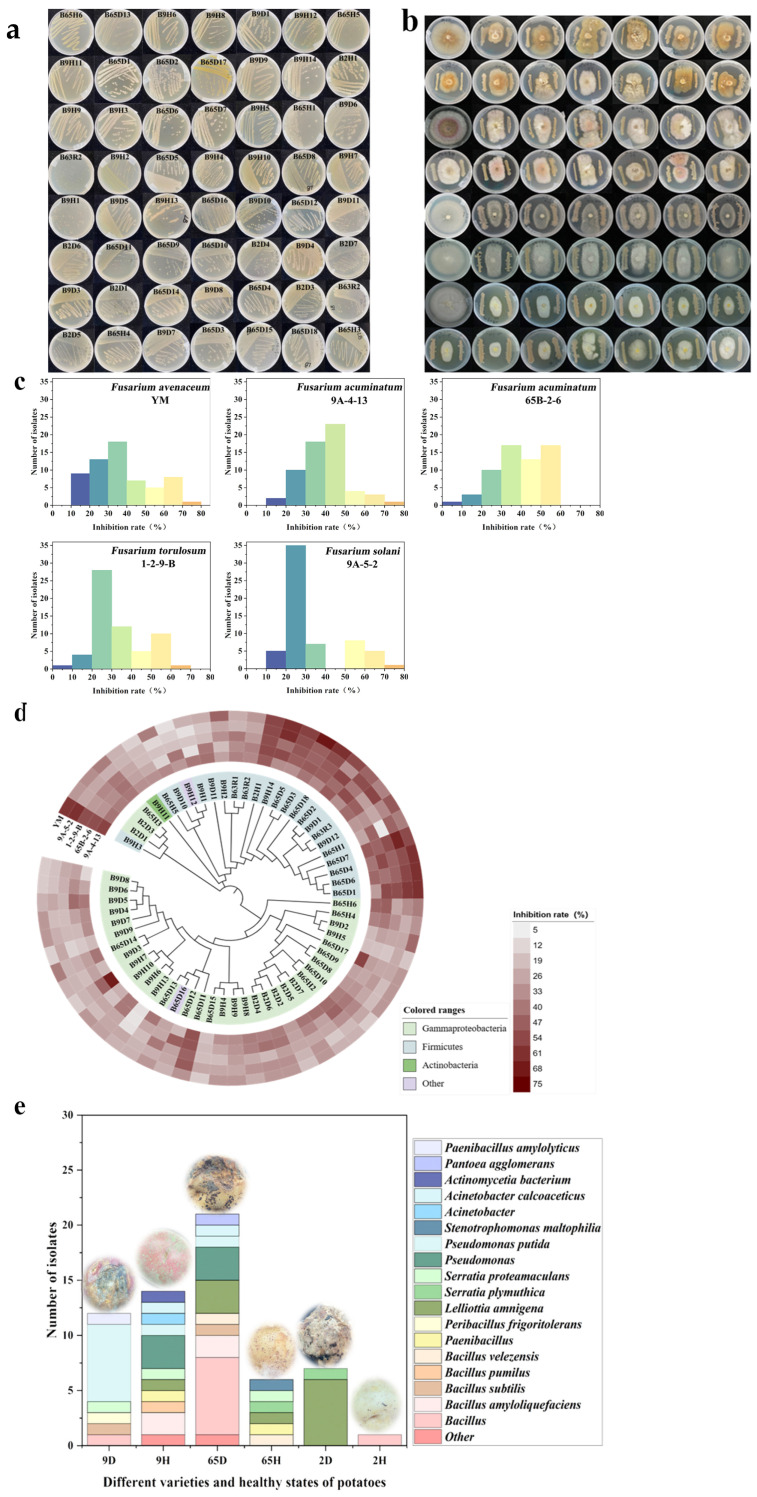
Cultivation of biocontrol strains for dry rot disease. (**a**,**b**) Morphology of isolated strains and their inhibition of *Fusarium* spp. (**c**) The range of inhibitory rates of isolated strains on *Fusarium* spp. (**d**) Phylogenetic tree of isolated strains and heat map of their inhibition rates against *Fusarium* spp. (**e**) Composition of strains isolated from potato samples of different varieties and healthy states (9D: Qingshu No. 9 diseased tuber, 9H: Qingshu No. 9 healthy tuber; 65D: Xiazhai No. 65 diseased tuber, 65H: Xiazhai No. 65 healthy tuber, 2D: Qingshu No. 2 diseased tuber, 2H: Qingshu No. 2 healthy tuber).

In total, 113 bacterial strains were isolated from potato samples with Qingshu No. 9 diseased (9D), Qingshu No. 9 healthy (9H), Xiazhai No. 65 diseased (65D), Xiazhai No. 65 healthy (65H), Qingshu No. 2 diseased (2D), and Qingshu No. 2 healthy (2H) (Figure 1a). Then, inhibitory activity of 61 strains against *Fusarium avenaceum*, *Fusarium torulosum*, *Fusarium solani*, and *Fusarium acuminatum* was screened (Figure 1b–d). The results showed that the inhibition rate of most strains was among 20–50% (Figure 1c). A study has found that more than 200 strains have been isolated, of which about 10% have high inhibitory activity against pathogens, about 10% have moderate inhibitory activity, and the remaining about 80% of strains have no significant reduction in disease phenotype [26]. This is consistent with our results.

In our experiment, 16s rDNA sequence analysis was performed on the 61 strains. The results showed that 29.51% of these 61 strains were *Bacillus* spp., including 6.56% *Bacillus amyloliquefaciens*, 3.28% *Bacillus subtilis*, 3.28% *Bacillus velezensis*, 1.64% *Bacillus pumilus*. Additionally, 24.59% of these 61 strains were *Pseudomonas* spp., including 14.75% *Pseudomonas putida*; 8.19% of these 61 strains were *Serratia* spp., including 1.64% *Serratia plymuthica*, 4.91% *Serratia proteamaculans*; 4.92% of these 61 strains were *Acinetobacter* spp., including 3.28% *Acinetobacter calcoaceticus*. Furthermore, these 61 strains include 18.03% *Lelliottia amnigena*, 3.28% *Paenibacillus* sp., 1.64% *Pantoea agglomerans*, 1.64% *Peribacillus frigoritolerans*, 1.64% *Stenotrophomonas maltophilia*, 1.64% *Actinomycetia bacterium*, 1.64% *Paenibacillus amylolyticus*, 3.28% others. The results indicated that Firmicutes is a highly inhibitory bacterial cluster against *Fusarium* spp. (Figure 1d). In addition, compared to other samples, 65D contains abundant *Bacillus* (Figure 1e). *Bacillus* has been proven to have significant advantages in inhibiting various plant pathogens, as it can enhance inhibitory activity through synergistic effects with other beneficial microorganisms [14]. Furthermore, Qingshu No. 9 is difficult to be infected by pathogens, and a large amount of *Pseudomonas putida* has been isolated from 9D. Research has found that a higher proportion of antagonistic microorganisms can be obtained from resistant plants [17]. Therefore, *Pseudomonas putida* should be primarily used for the construction of subsequent synthetic microbial communities. Studies also have demonstrated the potential of *Pseudomonas* in regulating plant growth and inhibiting diseases, due to the production of a large amount of antimicrobial secondary metabolites [8].

### 2.2. Isolation and Pathogenicity Assay of Pathogens for Dry Rot Disease

**Figure 2:**
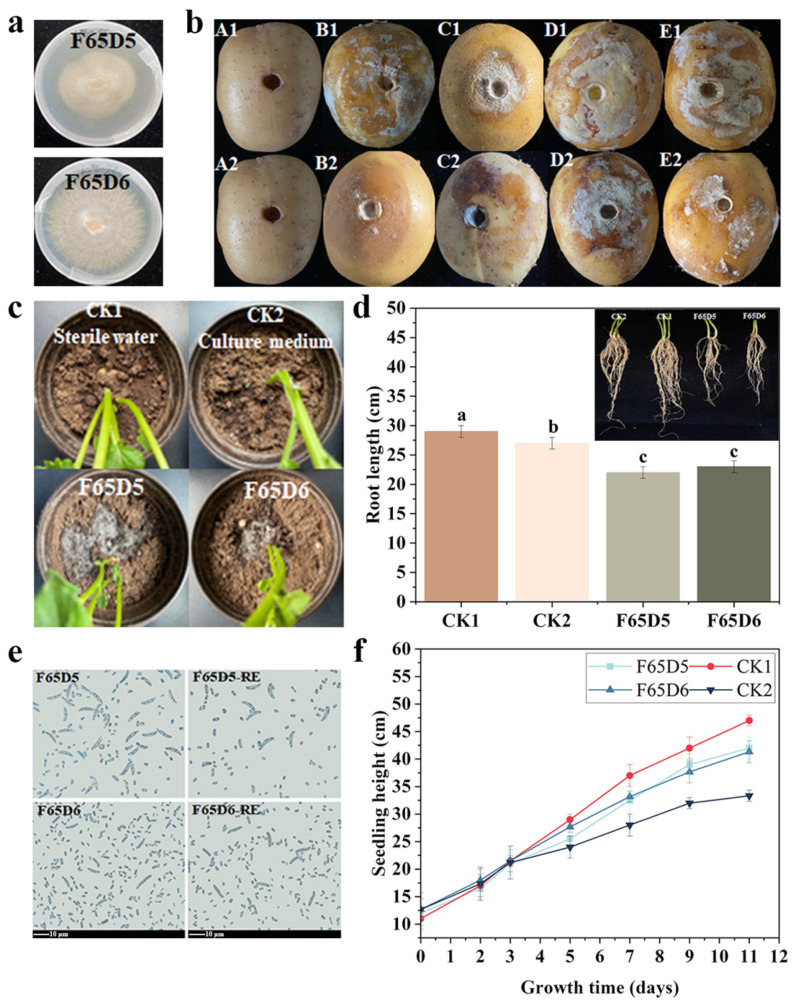
Cultivation of pathogenic strains for dry rot disease. (**a**) Morphology of isolated strains. (**b**) Pathogenicity assay of F65D5 and F65D6 strains (A1, A2: control, B1, B2: symptoms after inoculation of F65D5 and F65D6 plugs, C1, C2: symptoms after inoculation of F65D5 and F65D6 fermentation broth, D1, D2: symptoms after inoculation of F65D5 and F65D6 cell suspension, E1 and E2: symptoms after inoculation of F65D5 and F65D6 strains isolated again according to Koch’s rules). (**c**) The effect of inoculation with F65D5 and F65D6 strains on potted soil. (**d**) The effects of F65D5 and F65D6 on the root length of potato seedlings.The lowercase letters in (**d**) are significant difference markers (significance level is 0.05). (**e**) The spore morphology of F65D5 and F65D6 isolated for the first and second time follows Koch’s rule (the scale bar is 10 μm). (**f**) The effect of F65D5 and F65D6 on the height of potato seedlings.

A total of 33 fungal strains were isolated from potato samples of 65D, 65H, 9D, 9H, 2D, and 2H. After preliminary morphological observation, 12 fungal strains were selected to be identified for ITS sequence analysis. The results showed that *Fusarium solani* and *Fusarium oxysporum* accounted for 50% and 25% of the total isolated strains, respectively. Pathogenicity assays were conducted on *Fusarium solani* (F65D5) and *Fusarium oxysporum* (F65D6). When the fungal plug, fermentation broth and cell suspension of F65D5 and F65D6 strains infected potato tubers for 7 days, the results showed that F65D5 and F65D6 strains had completely infected potato tubers. And the disease intensity is highest after infection with cell suspension. Pathogens were isolated again from potato tubers infected with F65D5 and F65D6 diseases and inoculated into potato tubers, and it was found that they still infect healthy potatoes (Figure 2a,b). Therefore, F65D5 and F65D6 are pathogenic to potato tubers. Compared to F65D6, F65D5 strain has a higher infectivity. In addition, after inoculation with F65D5 and F65D6 in potato pots, white mycelium appeared in the soil (Figure 2c). F65D5 and F65D6 also have inhibitory effects on potato root length and seedling height (Figure 2d,f). Therefore, F65D5 and F65D6 are pathogenic to potato plants.

### 2.3. Screening of Synthetic Microbial Communities with Inhibitory Effects on Potato Dry Rot Disease

**Figure 3:**
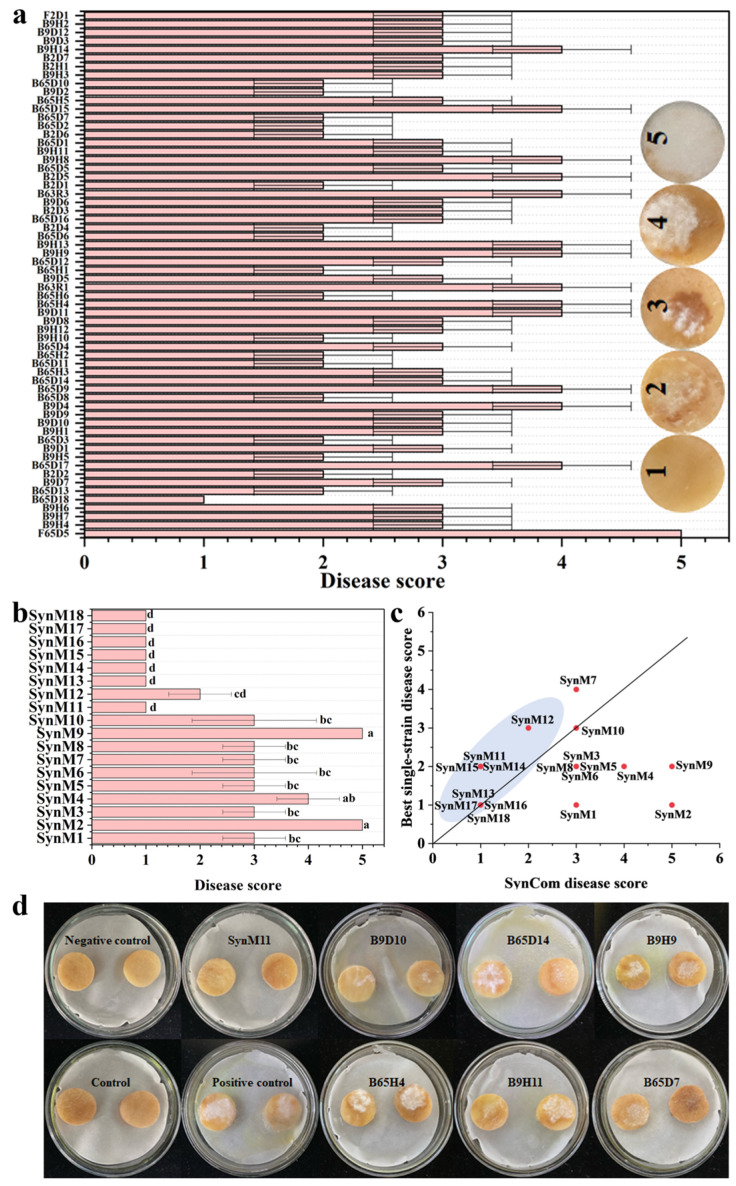
Preliminary screening of synthetic microbial communities. (**a**) Disease scores of all member strains of the synthetic microbial community. Disease score 1: No obvious symptoms of mycelium infection, Disease score 2: The area of mycelium infection is less than 15%, Disease score 3: The area of mycelium infection is less than 30%, Disease score 4: The area of mycelium infection is less than 45%, Disease score 5: The area of mycelium infection is more than 60%. (**b**) Disease score of synthetic microbial communities. The letters in represent significant difference markers (significance level is 0.05). (**c**) The disease score of synthetic microbial communities and the disease score of the best member strains in synthetic microbial communities (grey circle: the disease score of the best member of the synthetic community is higher than or equal to the disease score of the synthetic community). (**d**) The biocontrol effects of SynM11 and its member strains.

In order to screen for synthetic microbial communities with inhibitory activity against potato dry rot disease, 18 synthetic microbial communities were constructed (Table 1). A preliminary screening of inhibitory activity was conducted on synthetic microbial communities on ex vivo potatoes. The disease scores of SynM13, SynM16, SynM17, and SynM18 are all 1 point (Figure 3b), indicating that these synthetic microbial communities have high inhibitory activity. It can clearly be seen that the disease scores of SynM11, SynM12, SynM14, and SynM15 are lower than the disease scores of any of their members (Figure 3a–d). A study has shown that the synthetic microbial community established by *Bacillus*, *Paenibacillus*, *Streptomyces* has stronger inducible systemic resistance to *R. solanacearum* than its member strains [13]. This is consistent with our results (SynM11, SynM12, SynM14, and SynM15). Furthermore, the results showed that compared to synthetic microbial communities composed of the same genus, cross-genus synthetic microbial communities had lower disease scores (Figure 3b). Previous studies have shown that synthetic microbial communities composed of cross fungi and bacteria are more effective than individual fungal or bacterial synthetic microbial communities in inhibiting *Fusarium* wilt disease [28]. The reason may be that inhibiting diseases is the result of collective activity of microbial communities, and highly diverse microbial communities can more effectively trigger disease resistance [28,29]. Furthermore, our results showed that the number of members of the synthetic microbial community was not related to disease scores (Figure 3b). Previous studies have shown that large synthetic microbial communities (44 isolates) or small synthetic microbial communities (seven isolates) provide similar disease control [30]. Perhaps because the more microorganisms enriched in plants, the higher the resource cost required to maintain microbial growth and reproduction, and the presence of a large number of strains in the community may increase competition between them [8]. Based on the above results, we will further screen synthetic microbial communities with better inhibitory effects from SynM11, SynM12, SynM13, SynM14, SynM15, SynM16, SynM17, and SynM18.

**Figure 4:**
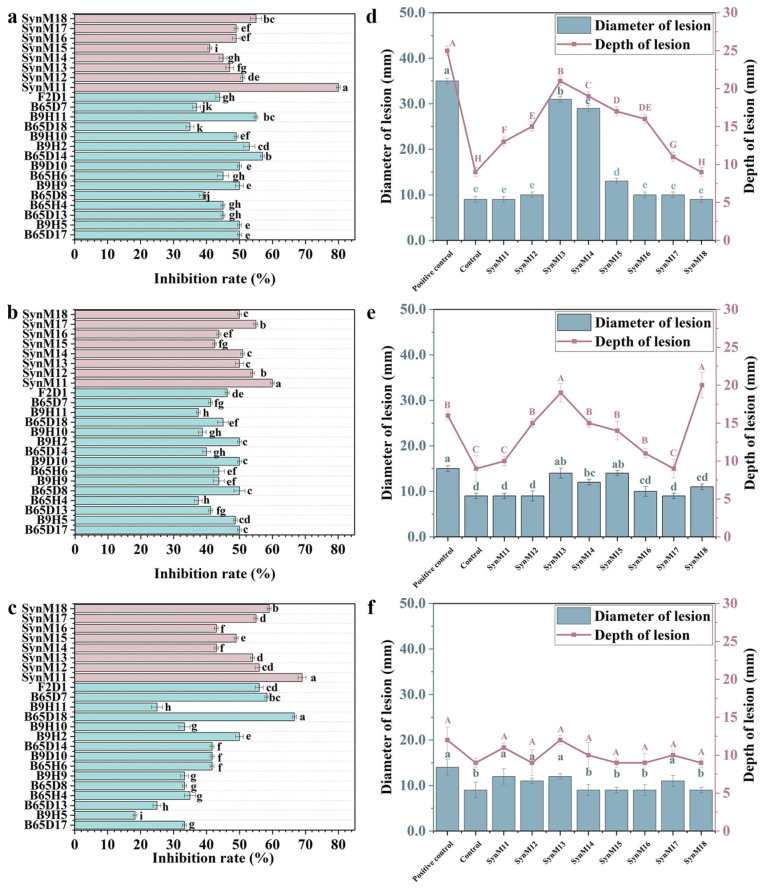
Re-screening of synthetic microbial communities. (**a**–**c**) The inhibition rates of synthetic microbial communities and their member strains on *Fusarium solani*, *Fusarium oxysporum*, and *Fusarium avenaceum* on vitro. Red represent synthetic communities, green represent single strains. (**d**–**f**) The effects of synthetic microbial communities on the diameter and depth of potato lesions infected with *Fusarium solani*, *Fusarium oxysporum*, and *Fusarium avenaceum*, respectively. The uppercase and lowercase letters in (**d**–**f**) represent significant difference markers (significance level is 0.05).

The inhibition rates of SynM11, SynM12, SynM13, SynM14, SynM15, SynM16, SynM17, and SynM18 against *Fusarium solani*, *Fusarium oxysporum*, and *Fusarium avenaceum* were tested in vitro. The results showed that SynM11 had an 80.00% inhibition rate against *Fusarium solani*, and its inhibitory activity was stronger than that of every member of the synthetic microbial community (Figure 4a–c). In addition, the optimal synthetic microbial community was screened on the whole potato tuber. The results showed that the infection intensity of different *Fusarium* spp. diseases varied. The infection of *Fusarium solani* is the strongest, while the infection of *Fusarium avenaceum* is the weakest (Figure 4d–f). Therefore, the next focus will also be studying on *Fusarium solani* as the target pathogen. After infection with *Fusarium solani*, SynM11, SynM12, and SynM18 have the best inhibitory activity. The lesion diameters of SynM11, SynM12, and SynM18 were 9 mm, 10 mm, and 9 mm, respectively. The depth of the lesion is 13 mm, 15 mm, and 9 mm, respectively (Figure 4d–f). In summary, through in vivo, in vitro, and detection of different pathogens, SynM11 was ultimately selected as subsequent researching target object (Table 2).

**Figure 5:**
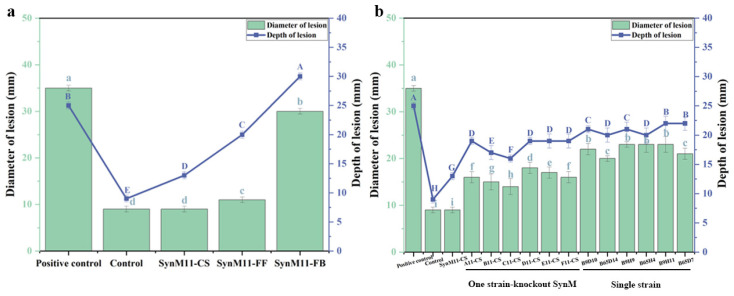
Screening of active components and member strains of synthetic microbial communities. (**a**) The effects of SynM11 cell suspension (SynM11-CS), fermentation filtrate (SynM11-FF), and fermentation broth (SynM11-FB) on the lesion diameter and depth of *Fusarium solani*-infected potatoes. (**b**) Synthetic communities, one strain-knockout synthetic communities, single strain affects the lesion diameter and depth of *Fusarium solani*-infected potatoes. The uppercase and lowercase letters in subfigures represent significant difference markers (significance level is 0.05).

On the other hand, the effects of SynM11 cell suspension (SynM11-CS), SynM11 fermentation filtrate (SynM11-FF), and SynM11 fermentation broth (SynM11-FB) on potato tuber diameter and depth damage caused by *Fusarium solani* were tested. The results showed that SynM11-CS had the best inhibitory activity against *Fusarium solani*, with a lesion diameter and depth of 9 mm and 13 mm, respectively. The lesion diameter and depth were similar to those of the control. And the lesion diameter and depth of SynM11-FB were 30 mm, 30 mm, respectively, which further damaged the potato tubers (Figure 5a).

Previous research has mainly focused on the overall function of synthetic microbial communities. However, the functions required for the synthetic microbial communities may mainly be attributed to individual microorganisms or additive effects involving all members [14]. A study has found that a small number of microbial members in synthetic microbial communities may play a crucial role in maintaining microbial community function [8]. Compared to the complete SynM11, the absence of any member in SynM11 will increase the diameter and depth of the lesion. Potato has the largest lesion diameter and depth after B65H4 being knocked out. And the inhibitory activity of B65H4 is not optimal when acting alone, which indicates that B65H4 is a key strain in SynM11 and also has an important relationship with SynM11 (Figure 5b). Therefore, it is necessary to explore the key role of B65H4 strain in the synthetic microbial community furtherly. Studies have shown that individual strains of Proteobacteria (*Sphingomonas*, *Rhizobia*) and Actinobacteria (*Microbacterium*, *Rhodococcus*) are most likely to affect community structure as key species [31].

### 2.4. Analysis of Active Metabolites in Synthetic Microbial Communities with Inhibitory Activity on Dry Rot Disease

**Figure 6:**
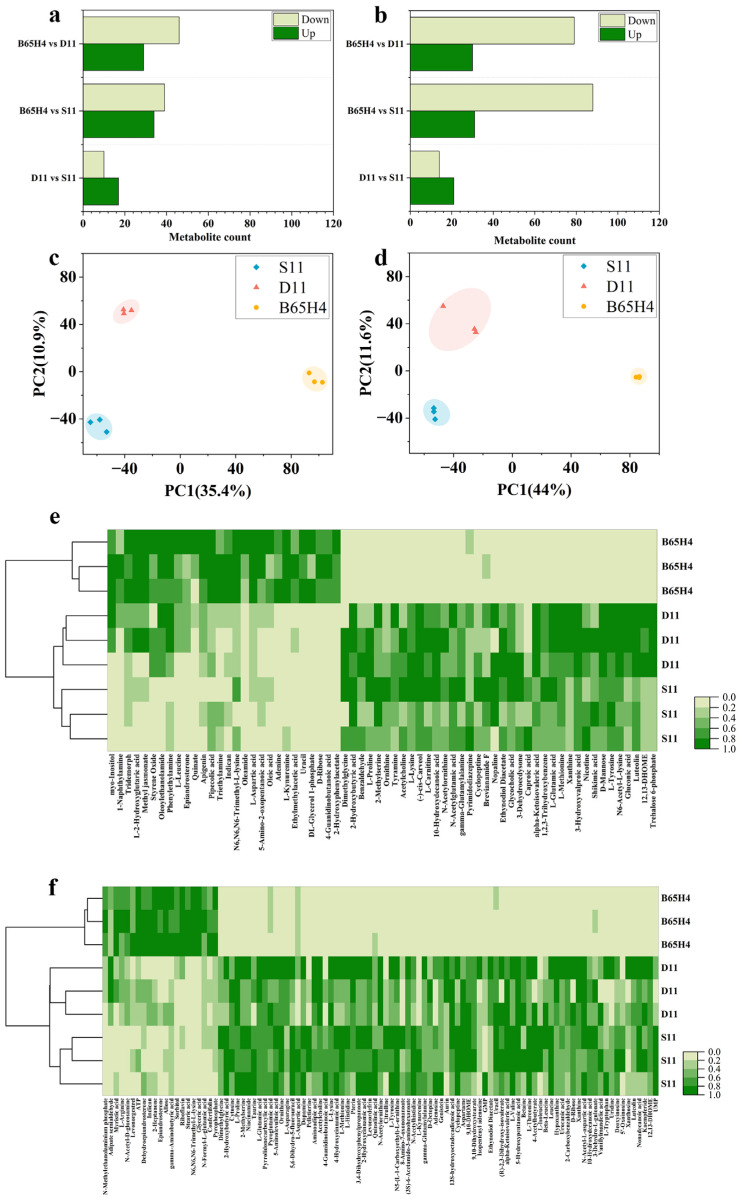
Comparative metabolomics of the synthetic microbial community, one strain-knockout synthetic communities, and the single strain. (**a**,**b**) The statistics of the quantity of extracellular (**a**) and intracellular (**b**) different metabolites (S11: SynM11, D11: SynM11 knockout B65H4 single strain, B65H4: single strain). (**c**,**d**) PLS-DA analysis of extracellular (**c**) and intracellular (**d**) different metabolites between S11, D11, and B65H4. (**e**,**f**) The heatmaps of extracellular (**e**) and intracellular (**f**) different metabolites between S11, D11, and B65H4.

Research suggests that secondary metabolites produced in synthetic microbial communities are the reason for their biocontrol activity [13]. The synthetic microbial community forms a complex network of beneficial species symbiosis, enhancing disease inhibition by secreting antimicrobial compounds [28]. The results in part 2.3 showed that B65H4 is a key strain in SynM11. Therefore, further detection of the metabolic profiles of SynM11 (S11), SynM11 knockout B65H4 single strain (D11), and B65H4 (single strain) was performed. The results showed that there were 75 extracellular and 109 intracellular different metabolites between B65H4 and D11, respectively. There are 73 extracellular and 119 intracellular different metabolites between B65H4 and S11, respectively. There are 27 extracellular and 35 intracellular different metabolites between D11 and S11, respectively (Figure 6a,b). According to the PLS-DA (partial least squares discriminant analysis) results, the S11, D11, and B65H4 groups can be clearly distinguished, indicating significant differences in metabolites between these three groups (Figure 6c,d). In addition, after comparing the intracellular and extracellular different metabolites between groups S11, D11, and B65H4, the results showed that brevianamide F, caproic acid, L-aspartic acid, and L-valve were enriched in S11 (Figure 6e,f). Brevianamides, a class of indole alkaloids, were isolated from *P. brevicompactum* in 1969 for the first time. Most of them exhibited anti-bacterial, anti-insect pests and anti-tubercular potentials [32,33]. In addition, organic acids can recruit beneficial microorganisms to resist *Fusarium* disease [28]. And specific organic acids are related to the improvement the colonization of *Pseudomonas* in tomato roots [34]. Furthermore, research has shown that caproic acid affects the mycelial growth inhibition rates of *A. solani* and *F. oxysporum* were 11.36% and 36.54%, respectively [35]. And the inhibitory rates of volatile organic compounds produced by *Pseudomonas fluorescens* on the growth mycelium and the conidial germination are 42.14% and 77.86%, respectively [36]. Moreover, L-aspartic acid play a crucial role in inhibiting the growth of pathogens and the production of fungal toxins [8]. Therefore, the metabolites speculated above are likely to be active metabolites that inhibit *Fusarium* spp. However, the compounds produced by synthetic microbial communities may be very complex and require further research [28].

### 2.5. Validation of Key Metabolites in Synthetic Microbial Communities

**Figure 7:**
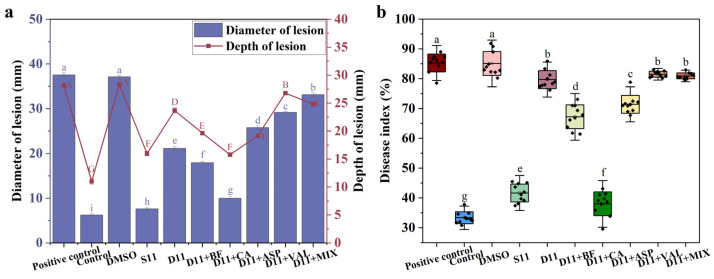
The effects of key metabolites on lesion diameter, lesion depth, and disease index of *Fusarium solani*-infected potatoes. (**a**) Lesion diameter and depth. (**b**) Disease index. (S11: SynM11, D11: SynM11 knockout B65H4 single strain. BF: brevianamide F, CA: caproic acid, ASP: L-aspartic acid, VAL: L-valve, MIX: mixing four key metabolites. DMSO: dimethyl sulfoxide). The uppercase and lowercase letters in subfigures represent significant difference markers (significance level is 0.05).

Brevianamide F, caproic acid, L-aspartic acid, and L-valve were enriched in S11(Figure 6e,f). Therefore, it is speculated that these four metabolites are key metabolites for S11 to produce high biocontrol activity. It is necessary to verify whether the key metabolites are active substances that inhibit *Fusarium solani*. The lesion diameter, lesion depth, and disease index of potatoes infected by *Fusarium solani* after the addition of brevianamide F, caproic acid, L-aspartic acid, and L-valve were tested. The results showed that after adding caproic acid, the diameter and depth of the lesion were the lowest, at 10 mm and 16 mm, respectively (Figure 7a). The disease index is also the lowest, at 38.01% (Figure 7b). Therefore, further research on the biocontrol mechanism of caproic acid is needed.

### 2.6. The Biocontrol Mechanism of Caproic Acid

**Figure 8:**
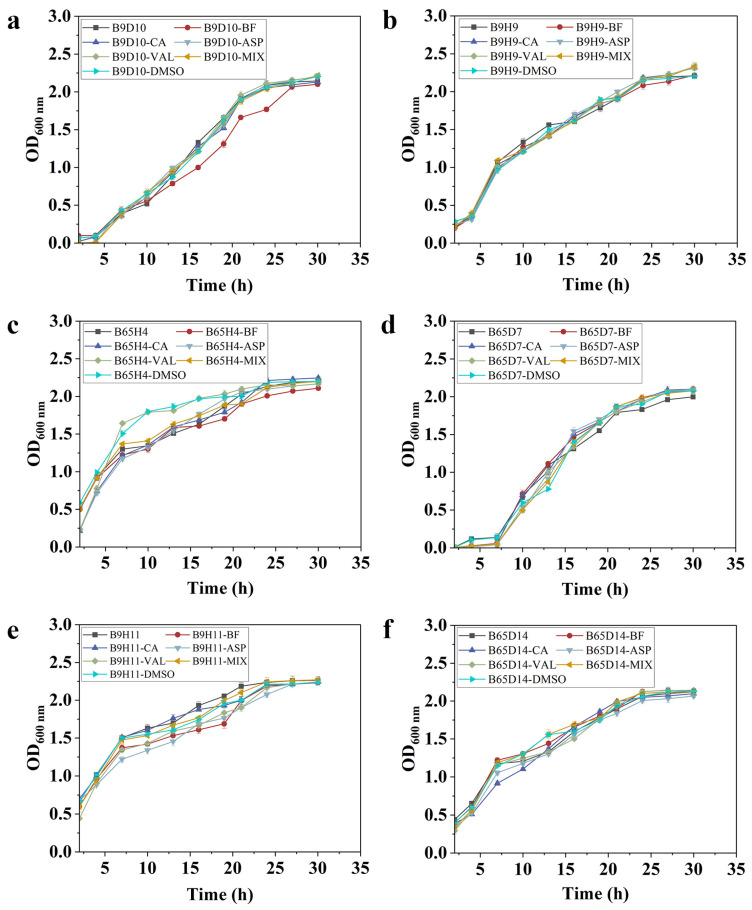
The effect of key metabolites on the growth of member strains of synthetic microbial communities. (**a**) B9D10. (**b**) B9H9. (**c**) B65H4. (**d**) B65D7. (**e**) B9H11. (**f**) B65D14. (BF: breviamide F. CA: caproic acid. ASP: L-aspartic acid. VAL: L-valve. MIX: mixing these four metabolites. DMSO: dimethyl sulfoxide).

It is important to detect the activity mechanism of caproic acid from the perspective of the synthetic microbial community members. The effects of key metabolites (breviamide F, caproic acid, L-aspartic acid, and L-valve) on the growth of synthetic microbial community member strains were tested. The results showed that none of these four key metabolites had a significant impact on the growth of member strains of the synthetic microbial community (Figure 8a–f). This indicates that the key metabolites meet the basic requirements of biocontrol, that is, they do not inhibit the growth of biocontrol strains to further exert biocontrol activity. Studies have shown that the proteins and volatile compounds secreted by *Trichoderma* significantly inhibit the growth of tomato rhizosphere bacteria. Therefore, it is proposed that this may affect plant health and the effectiveness of biocontrol [37]. Previous studies have also shown that metabolites of synthetic microbial communities promote the growth of members of synthetic microbial communities [14]. The reason may be that previous studies used fermentation filtrates from synthetic microbial communities for detection, while in this study, we used a single metabolite to detect the impact on the growth of member strains. Furthermore, a study suggests that metabolic interactions are highly dependent on the environment, and syntrophic cooperation only occurs in statically nutrient rich niches [38]. Therefore, we need to further explore the impact of key metabolites on the growth of synthetic microbial community members in more complex environments.

**Figure 9:**
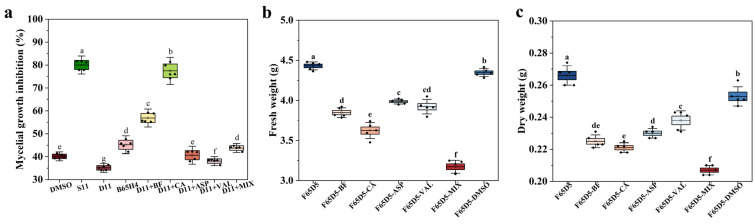
The effects of key metabolites on *Fusarium solani* mycelium. (**a**) The effect of key metabolites on the inhibition rate of mycelial growth. (**b**,**c**) The effect of key metabolites on the fresh and dry weight of mycelium. (S11: SynM11, D11: SynM11 knockout B65H4 single strain, B65H4: the single strain. F65D5: *Fusarium solani*. BF: breviamide F. CA: caproic acid. ASP: L-aspartic acid. VAL: L-valve. MIX: mixing these four metabolites. DMSO: dimethyl sulfoxide). The lowercase letters in subfigures represent significant difference markers (significance level is 0.05).

On the other hand, biocontrol agents enhance their effectiveness by interacting with pathogens [39,40]. The effects of breviamide F, caproic acid, L-aspartic acid, and L-valve on the mycelium of *Fusarium solani* were tested. The results showed that caproic acid had the highest inhibition rate on the mycelial growth of *Fusarium solani*, reaching 77.54% (Figure 9a). After treatment with caproic acid, the fresh and dry weight of *Fusarium solani* mycelium were also the lowest, at 3.628 g and 0.221 g, respectively (Figure 9b). The mycelium of *Fusarium solani* has an important function in infecting potatoes and causing disease [41]. SynM11 may inhibit *Fusarium solani* mycelium by metabolizing caproic acid, thereby inhibiting potato dry rot disease. 

**Figure 10:**
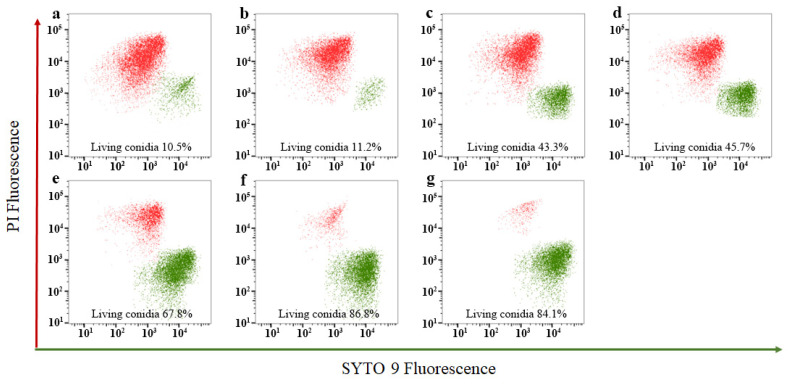
Flow cytometry analysis of the effects of key metabolites on the conidia viability of *Fusarium solani.* (**a**) Positive controls. (**b**) *Fusarium solani* treated with caproic acid. (**c**) *Fusarium solani* treated with brevianamide F. (**d**) *Fusarium solani* treated with mixed metabolite. (**e**) *Fusarium solani* treated with L-ASP. (**f**) *Fusarium solani* treated with L-VAL. (**g**) Negative control.

The classic plating technique is often used to detect conidia viability, but it cannot provide detailed real-time information about the physiological status of conidia [42]. Flow cytometry and fluorescence microscopy can sensitively distinguish between live and dead cells. The effects of four key metabolites on the viability of *Fusarium solani* conidia were tested by flow cytometry. The results showed that compared with other key metabolites, the treatment of *Fusarium solani* conidia with caproic acid resulted in the lowest living conidia rate, which was 11.2% (Figure 10a–g). This further demonstrates the inhibitory activity of caproic acid metabolized by the synthetic microbial community against *Fusarium solani*. This is also consistent with the previous inhibitory effect of caproic acid on *Fusarium solani* mycelium.

**Figure 11:**
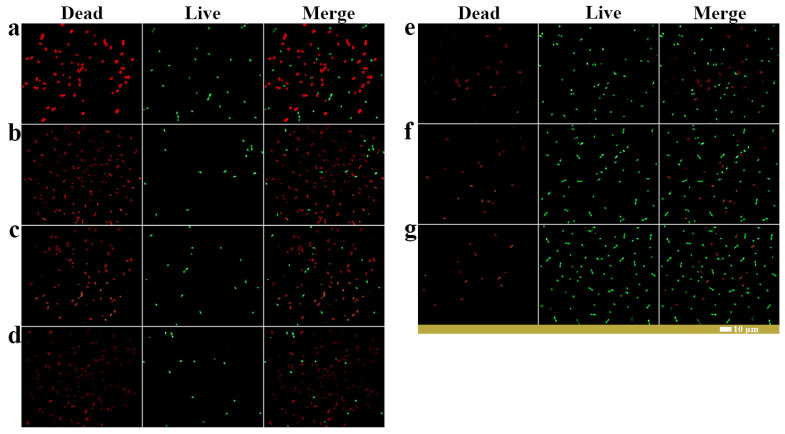
Fluorescence microscopy images of the effects of key metabolites on the growth of *Fusarium solani* conidia. (**a**) Positive controls. (**b**) *Fusarium solani* treated with caproic acid. (**c**) *Fusarium solani* treated with brevianamide F. (**d**) *Fusarium solani* treated with mixed metabolite. (**e**) *Fusarium solani* treated with L-ASP. (**f**) *Fusarium solani* treated with L-VAL. (**g**) Negative control. Red represents dead conidia, green represents live conidia.

The fluorescence microscope results showed that compared with other metabolite treatments, the conidia of *Fusarium solani* treated with caproic acid showed stronger red fluorescence and weaker green fluorescence. This more intuitively indicates that caproic acid has an inhibitory effect on the conidia of *Fusarium solani* (Figure 11a–g). In summary, SynM11 directly inhibits the growth of *Fusarium solani* mycelium and conidia by metabolizing high concentrations of caproic acid, ultimately achieving a biocontrol effect on potato dry rot disease. However, biocontrol agents can not only inhibit the growth of fungal mycelia and conidia germination, but also affect DNA processing enzymes and repair mechanisms by binding to pathogen DNA [43,44]. In future research, we will further explore the accumulation mode of caproic acid in the synthetic microbial community and its deeper inhibitory mechanism on *Fusarium solani* (Figure 2 and Table 2).

## 3. Materials and Methods

### 3.1. Isolation of Microorganisms and Evaluation of Antifungal Activity

Samples of different potato varieties (Qingshu No. 9, Xiazhai No. 65, Qingshu No. 2), physiological positions (surface and endophytic parts of potatoes), and physiological states (healthy and diseased potatoes) were collected. The potato samples were ground using a sterile mortar and pestle. Then continuously dilute the supernatant and apply the obtained supernatant to the agar plate for cultivation. Using five different agar plate to isolate strains (LB, YPD, Martini’s, Gause’s No. 1, and PDA culture mediums, respectively). The isolated strains were continuously purified for 2–3 generations [45]. Furthermore, *Fusarium avenaceum* (YM), *Fusarium torulosum* (1-2-9-B), *Fusarium solani* (9A-5-2), and *Fusarium acuminatum* (65B-2-6, 9A-4-13) strains all come from strains previously preserved in our laboratory.

An active growing *Fusarium* spp. (*Fusarium avenaceum*, *Fusarium torulosum*, *Fusarium solani*, *Fusarium oxysporum*, *and Fusarium acuminatum*) plug was inoculated in the center of the PDA plate. Then we inoculated the isolated strains on both sides of the *Fusarium* spp. plug. *Fusarium* spp. were inoculated onto PDA plates as a control group. The plates were incubated at 28 °C for 7 days. The diameter of mycelial growth was measured as the average of two perpendicular measurements [46]. We calculated the inhibition rate according to the following formula.

(1) $$\mathrm{Inhibition}\;\mathrm{rate}\;(\%)=\frac{{\mathrm{Diameter}}_{\mathrm{control}\;\mathrm{mycelium}}-{\;\mathrm{Diameter}}_{\mathrm{mycelium}\;\mathrm{after}\;\mathrm{treatment}}}{{\mathrm{Diameter}}_{\mathrm{control}\;\mathrm{mycelium}}}\;\times \;100$$

### 3.2. Identification of Microorganisms

The bacterial genomic DNA was extracted and the 16S rDNA gene sequence of the isolated strains using primers 27F (5′-AGAGTTTGATCCTGGCTCAG-3′) and 1492R (5′-GGTTACCTTGTTACGACTT-3′) was amplified. Similarly, we extracted the fungal genomic DNA and amplified the ITS gene sequence of the isolated strains using primers ITS1 (5′-GGTTTTGATCCTTGTCTCCAG-3′) and ITS4 (5′-GGTTACCTGTTACGACTT-3′). PCR amplification products were detected by 0.5% agarose gel electrophoresis and sequenced after passing the detection. We then submitted the sequencing results to the GenBank database of NCBI, performed homologous alignment with known sequences using BLAST, and constructed a phylogenetic tree using MEGA version 11.0 software [47].

### 3.3. Pathogenicity Assay of Isolated Strains

Pathogenicity assay of potato tubers: We made a uniform wound at the midpoint of each disinfected potato, inoculated with pathogen plug; 50 μL, 1.0 × 10^7^ conidia/mL pathogen fermentation broth and cell suspension were used, with wounds treated with 10 mM MgCl_2_ as the control. It was cultured in an incubator at 30 °C and 75% humidity for 14 days. The condition of the potato tubers was observed daily. Pathogenicity assay of potato potted plants: We planted the sprouted potato tubers in potted soil, and when the seedlings reached the appropriate height, we chose a potato potted plant with the same seedling height and physiological condition. We watered 200 mL of pathogen fermentation broth with OD_600nm_ =1 along the roots of potato seedlings. We observed and detected the potato seedling height and root length [48,49].

### 3.4. Constructing and Screening Synthetic Microbial Communities

We cultivated the member strains of the synthetic microbial community in LB medium for 18 h, with an OD_600nm_ of 0.8 for each member strain, and then mixed the same volume of each strain into a centrifuge tube. We centrifuged the synthetic microbial community in a centrifuge tube at 4 °C, 10,000 r/min for 10 min. We obtained the fermentation filtrate and cell of the synthetic microbial community, then suspended the cell in a 10 mM MgCl_2_ solution to prepare a cell suspension. We adjusted the concentration of synthetic microbial community cells to 1.0 × 10^7^ cfu/mL.

Prevention experiment of potato dry rot disease: first we added 20 μL 1.0 × 10^7^ cfu/mL synthetic microbial community to disinfected potatoes, cultured for 24 h and inoculated with 20 μL 1.0 × 10^7^ conidia/mL *Fusarium* spp. cell suspension. The mixture was cultured in an incubator at 30 °C and 75% humidity, and then we assessed the disease score (1–5 points), or measured the diameter and depth of lesions. Disease score 1: No obvious symptoms of mycelium infection, Disease score 2: The area of mycelium infection is less than 15%, Disease score 3: The area of mycelium infection is less than 30%, Disease score 4: The area of mycelium infection is less than 45%, Disease score 5: The area of mycelium infection is more than 60%. 

We placed a *Fusarium* spp. plug at the center of the agar plate, then placed four pieces of drug-sensitive paper around the same distance as the *Fusarium* spp. plug. We added 1.0 × 10^7^ cfu/mL, 10 μL synthetic microbial communities to the drug-sensitive paper, and incubated it at 28 °C for 7 days to test the in vitro inhibition rate of the synthetic microbial community. The calculation formula is the same as shown in Section 3.1.

### 3.5. Analysis and Validation of Active Metabolites in Synthetic Microbial Communities

Comparative metabolomics experimental samples: The constructed synthetic community (S11), the single strain knockout synthetic community (D11), and the key single strain (B65H4). The preparation method of the above samples is described in Section 3.4. Sample treatment: we thawed the above experimental sample at 4 °C, vortexed the sample for 1 min after thawing, and mixed evenly. We accurately transferred an appropriate amount of sample into a 2 mL centrifuge tube, concentrated and dried it. We added 500 µL methanol solution into the sample tube dried in step 2 and vortexed for 1 min. We centrifuged it for 10 min at 12,000 rpm and 4 °C, took all the supernatant, transferred it to a new 2 mL centrifuge tube, concentrated and dried it. We added 150 µL of 2-Amino-3-(2-chloro-phenyl)-propionic acid (4 ppm) solution prepared with 80% methanol water to redissolve the sample, filtered the supernatant by 0.22 μm membrane and transferred into the detection bottle for LC-MS detection [50,51].

Liquid chromatography conditions: the column was maintained at 40 °C. The flow rate and injection volume were set at 0.25 mL/min and 2 μL, respectively [52]. For LC-ESI (+)-MS analysis, the mobile phases consisted of (B2) 0.1% formic acid in acetonitrile (*v*/*v*) and (A2) 0.1% formic acid in water (*v*/*v*). Separation was conducted under the following gradient: 0~1 min, 8% B2; 1~8 min, 8%~98% B2; 8~10 min, 98% B2; 10~10.1 min, 98%~8% B2; 10.1~12 min, 8% B2. For LC-ESI (-)-MS analysis, the analytes were carried out with (B3) acetonitrile and (A3) ammonium formate (5 mM). Separation was conducted under the following gradient: 0~1 min, 8% B3; 1~8 min, 8~98% B3; 8~10 min, 98% B3; 10~10.1 min, 98%~8% B3; 10.1~12 min, 8% B3. Mass spectrum conditions: mass spectrometric detection of metabolites was performed on Q Exactive (Thermo Fisher Scientific, USA) with ESI ion source. The parameters were as follows: sheath gas pressure, 30 arb; aux gas flow, 10 arb; spray voltage, 3.50 kV and −2.50 kV for ESI (+) and ESI (−), respectively; capillary temperature, 325 °C; MS1 range, *m*/*z* 100–1000; MS1 resolving power, 70,000 FWHM; number of data-dependent scans per cycle, 3; MS/MS resolving power, 17,500 FWHM; normalized collision energy, 30 eV; dynamic exclusion time, automatic [53].

To verify active metabolites: we added 100 μL 1.0 × 10^7^ cfu/mL D11 (SynM11 knockout B65H4 single strain) to potato tubers, then added 20 μL key metabolite. After 24 h of cultivation, we added 50 μL 1.0 × 10^7^ conidia/mL *Fusarium solani* cell suspension. It was cultured in an incubator at 30 °C and 75% humidity, and then we measured the diameter and depth of lesions. We calculated the disease index as previously reported [8].

### 3.6. Detecting the Growth of Bacterial Strains

We added synthetic microbial community member strains to LB liquid culture medium, then added 100 μL key metabolites to a shake flask culture, cultivated it at 28 °C and 180 r/min for 30 h and detected the growth of all strains according to previous literature reports [14].

### 3.7. Fungal Survival Assay

We inoculated a *Fusarium solani* plug in the middle of PDA culture medium, placed four pieces of drug-sensitive paper around it, and then added 20 μL D11, 4 μL key metabolites on top of it. After 7 days of cultivation at 28 °C, we calculated the mycelial growth inhibition rate according to the formula in Section 3.1. We prepared 50 mL of PDB sterile culture medium, then added 1 mL of key metabolites and 2 mL of *Fusarium solani* cell suspension at 28 °C for 5 days at 180 r/min. We measured the dry and fresh weight of the mycelium. Specifically, after filtering the mycelium, we used filter paper to absorb the moisture between the mycelium, and weighed it to obtain the fresh weight of the mycelium. After drying at 105 °C to a constant weight, we weighed it to obtain the dry weight of the mycelium [8].

### 3.8. Flow Cytometric and Fluorescence Microscopy Assessment of Conidial Viability

We added 1 mL of key metabolites to a 2 mL, 1.0 × 10^6^ conidia/mL *Fusarium solani* cell suspension. After incubation for 24 h, we washed the suspension twice in sterile saline (3000 r/min, 8 min, 4 °C) and finally resuspended in 997 μL of sterile saline. These samples were stained with 3 μL of a mixture of SYTO 9 (5 μM final concentration), PI (30 μM final concentration) and were incubated for 10 min in the dark at room temperature. Flow cytometry analysis was performed using Guava easy Cell Systems (EMD Millipore Corporation, Hayward, CA, USA). Observation was performed using a Zeiss inverted fluorescence microscope (Carl Zeiss AG, Oberkochen, Germany) [42].

### 3.9. Statistical Analysis

All statistical analyses were performed using Origin 2023. Data are presented as mean ± standard error (SE). Multiple comparisons were analyzed by one-way analysis of variance (ANOVA) followed by the least significant difference (LSD) multiple-range test and compared to demonstrate differences between the means using Tukey’s HSD test. Involving potato in vivo experiments were repeated three times, with 10 parallels set each time.

## 4. Conclusions

In summary, the interaction between *Serratia protoamaculans* and other member strains (*Paenibacillus amylolyticus*, *Pseudomonas putida*, *Acinetobacter calcoaceticus*, *Actinomycetia bacterium*, *Bacillus subtilis*) in the synthetic microbial community (SynM11) produces higher concentrations of caproic acid, which directly inhibits the occurrence of potato dry rot by inhibiting the mycelium and conidia of *Fusarium solani*. Furthermore, compared to the single strains, the biocontrol activity against potato dry rot disease was improved by synthesizing microbial communities. This study provides a new perspective on the application of synthetic microbial communities in potato dry rot disease. In the future, further research is needed to investigate the special contributions of each member in the synthetic microbial community to the biocontrol effect, and to deeply analyze the interaction relationship between each member strain.

## Figures and Tables

**Table 1:** Designed monobacteria and synthetic microbial communities for biocontrol groups.

Biocontrol Group		Distribution of Strain Species (Genera)
BM	Monobacteria	Distributed in 10 genera (*Bacillus*, *Paenibacillus*, *Peribacillus frigoritolerans*, *Lelliottia amnigena*, *Serratia proteamaculans*, *Pseudomonas*, *Acinetobacter*, *Actinomycetia bacterium*, *Paenibacillus amylolyticus*, *Pantoea agglomerans*)
SynM1	Bacterial synthesis community (61 strains)	Distributed in 10 genera (Synthesized from all strains in BM)
SynM2	Bacterial and fungal synthetic communities (62 strains)	Distributed in 11 genera (SynM1+*Penicillium allii*)
SynM3	*Bacillus* synthetic community (17 strains)	Distributed in five species (*Bacillus* sp., *Bacillus amyloliquefaciens*, *Bacillus subtilis*, *Bacillus pumilus*, *Bacillus velezensis*)
SynM4	*Lelliottia amnigena* synthetic community (11 strains)	Distributed in one species (*Lelliottia amnigena*)
SynM5	*Serratia* synthetic community (five strains)	Distributed in two species (*Serratia plymuthica*, *Serratia proteamaculans*)
SynM6	*Pseudomonas* synthetic community (15 strains)	Distributed in one species (*Pseudomonas putida*)
SynM7	*Acinetobacter* synthetic community (three strains)	Distributed in one species (*Acinetobacter calcoaceticus*)
SynM8	Bacterial synthesis community (16 strains)	Distributed in nine genera (*Bacillus* sp., *Bacillus amyloliquefaciens*, *Paenibacillus* sp., *Bacillus subtilis*, *Bacillus pumilus*, *Bacillus velezensis*, *Peribacillus frigoritolerans*, *Lelliottia amnigena*, *Serratia plymuthica*, *Serratia proteamaculans*, *Pseudomonas* sp., *Pseudomonas putida*, *Acinetobacter* sp., *Acinetobacter calcoaceticus*, *Actinomycetia bacterium*, *Paenibacillus amylolyticus*)
SynM9	Bacterial and fungal synthetic communities (17 strains)	Distributed in 10 genera (SynM8+*Penicillium allii*)
SynM10	Bacterial synthesis community (six strains)	Distributed in six genera (*Bacillus amyloliquefaciens*, *Serratia proteamaculans*, *Pseudomonas putida*, *Acinetobacter calcoaceticus*, *Actinomycetia bacterium*, *Paenibacillus amylolyticus*)
SynM11	Bacterial synthesis community (six strains)	Distributed in six genera (*Bacillus subtilis*, *Serratia proteamaculans*, *Pseudomonas putida*, *Acinetobacter calcoaceticus*, *Actinomycetia bacterium*, *Paenibacillus amylolyticus*)
SynM12	Bacterial synthesis community (six strains)	Distributed in six genera (*Bacillus pumilus*, *Serratia proteamaculans*, *Pseudomonas putida*, *Acinetobacter calcoaceticus*, *Actinomycetia bacterium*, *Paenibacillus amylolyticus*)
SynM13	Bacterial synthesis community (six strains)	Distributed in six genera (*Lelliottia amnigena*, *Bacillus velezensis*, *Pseudomonas* sp., *Serratia proteamaculans*, *Stenotrophomonas maltophilia*, *Pantoea agglomerans*)
SynM14	Bacterial and fungal synthetic communities (seven strains)	Distributed in seven genera (*Paenibacillus amylolyticus*, *Pseudomonas putida*, *Acinetobacter calcoaceticus*, *Serratia proteamaculans*, *Actinomycetia bacterium*, *Bacillus subtilis*, *Penicillium allii*)
SynM15	Bacterial and fungal synthetic communities (seven strains)	Distributed in seven genera (*Bacillus pumilus*, *Pseudomonas putida*, *Acinetobacter calcoaceticus*, *Serratia proteamaculans*, *Actinomycetia bacterium*, *Bacillus subtilis*, *Penicillium allii*)
SynM16	Bacterial and fungal synthetic communities (seven strains)	Distributed in seven genera (*Lelliottia amnigena*, *Bacillus velezensis*, *Pseudomonas* sp., *Serratia proteamaculans*, *Stenotrophomonas maltophilia*, *Pantoea agglomerans*, *Penicillium allii*)
SynM17	Bacterial synthesis community (five strains)	Distributed in five genera (*Bacillus velezensis*, *Pseudomonas* sp., *Serratia proteamaculans*, *Stenotrophomonas maltophilia*, *Pantoea agglomerans*)
SynM18	Bacterial and fungal synthetic communities (six strains)	Distributed in six genera (*Bacillus velezensis*, *Pseudomonas* sp., *Serratia proteamaculans*, *Stenotrophomonas maltophilia*, *Pantoea agglomerans*, *Penicillium allii*)

Note: The white areas represent the monobacteria biocontrol group, the gray areas represent the large synthetic microbial communities, the pink areas are the small to medium synthetic microbial community formed by the same genus, the yellow areas represent the medium synthetic microbial community, the green areas represent the small synthetic microbial communities.

**Table 2:** Member strains in SynM11.

No.	Member Strains in SynM11	Strains Name
1	B9D10	*Paenibacillus amylolyticus*
2	B65D14	*Pseudomonas putida*
3	B9H9	*Acinetobacter calcoaceticus*
4	B65H4	*Serratia proteamaculans*
5	B9H11	*Actinomycetia bacterium*
6	B65D7	*Bacillus subtilis*

## Data Availability

Data is contained within the article.
